# The Photocatalytic Activity of Titania Coatings Produced by Electrochemical and Chemical Oxidation of Ti6Al4V Substrate, Estimated According to ISO 10678:2010

**DOI:** 10.3390/ma13112649

**Published:** 2020-06-10

**Authors:** Michalina Ehlert, Aleksandra Radtke, Adrian Topolski, Julia Śmigiel, Piotr Piszczek

**Affiliations:** 1 Faculty of Chemistry, Nicolaus Copernicus University in Toruń, Gagarina 7, 87-100 Toruń, Poland; m.ehlert@doktorant.umk.pl (M.E.); topolski@umk.pl (A.T.); 279506@stud.umk.pl (J.Ś.); 2 Nano-Implant Ltd., Gagarina 5, 87-100 Toruń, Poland

**Keywords:** titania coatings, photocatalytic activity, band gap, methylene blue photobleaching effect, rate constants, ISO 10678:2010

## Abstract

The last twenty years have been a period of intense investigations of materials based on titanium dioxide, which have unique properties and functionalities, and which can be used in various areas of medicine. As a part of this issue, the results of our works for the assessment of the photocatalytic activity of titanium dioxide nanocoatings of different nanoarchitecture (nanoporous, nanotubular, nanosponge-like and nanofibrous examples), which were earlier checked in terms of their biocompatibility and usability for the modification of medical devices’ surfaces, are presented. The studied materials were produced on the surface of Ti6Al4V substrates using electrochemical and chemical oxidation methods. The activity of produced titania materials was studied on the base of the methylene blue (MB) degradation effect, in accordance to ISO 10678:2010. In our works, we have focused on the analysis of the correlation between the photocatalytic activity of nanoarchitecturally different TiO_2_ coatings, their morphology and structure. The obtained results prove that all studied coatings, both amorphous and amorphous containing crystalline domains, revealed photocatalytic activity in the photoinduced degradation of the organic pollution standard. This activity may be an additional advantage of medical device coatings, being adequate for use in sterilization processes applying UVA light.

## 1. Introduction

The high photocatalytic activity of titanium dioxide, its good chemical and thermal stability, as well as the relatively low cost of materials based on TiO_2_ production mean that they are used in the removal of environmental pollution (e.g., pesticides, drugs, hormones, exhaust gases) [1,2,3,4,5,6,7,8], or production of self-cleaning and self-sterilizing surfaces (e.g., the production of windows, car windows and mirrors) [1,2,3,9,10]. Another important application field of this type of materials is their use as agents against bacteria, fungi, viruses and even cancer cells. The use of titania nanoparticles to neutralize methicillin-resistant *Staphylococcus aureus* (MRSA), *Pseudomonas* bacteria and the influenza virus, which are responsible for serious diseases, is an example [11,12,13]. Nakamo et al. showed that, even with low UVA intensity and a short exposure time, a significant reduction in influenza viruses was observed. Similar effects were obtained when studying the interaction of MRSA with a photocatalytically active TiO_2_ nanosystem [11]. According to Chaturvedi and Dave [14], TiO_2_-based nanomaterials can also be used to remove toxic and carcinogenic gases, such as volatile organic compounds, which are thought to cause children’s blood cancers, from the air. The disinfectant properties of this type of materials are three times more effective than chlorine, and one and half times more than ozone. This effect is associated with the formation of reactive oxygen species (ROS), i.e., hydroxyl radicals, hydrogen peroxide, and superoxide anions, which are produced after TiO_2_ is exposed to UV light. The arising concentration of ROS on the surface of titania nanoparticles accelerates the breaking of chemical bonds in volatile organic compounds, which makes gases non-harmful to humans [14]. The titania nanoparticles’ photocatalytic activity has also been used in photodynamic anticancer therapy (PDT) [15]. This is a method of destroying cancer cells which uses the reaction between the photosensitiser (e.g., TiO_2_) and electromagnetic radiation of the appropriate wavelength (e.g., UVA).

The mechanism of the photocatalytic activity of titania nanoparticles or nanolayers of different architectures was widely studied and discussed in previous literature reports [14,16,17,18,19,20,21,22,23]. This mechanism assumes the excitation of their surface by photons whose energy is close to that of the band gap, and the creation of an electron-hole pair in result. The electrons of the conduction band in contact with adsorbed oxygen molecules form $O_{2}^{•-}$ superoxide anion radicals. At the same time, a positively charged surface (holes) acquires electrons from water adsorbed from the air, which leads to the formation of hydroxyl ^•^OH radicals. This mechanism leads to oxidation and reduction reactions which occur on the surface of titania coatings and which are responsible for the strong biological activity of this type of material [1,16,22,23,24,25,26]. 

Titanium dioxide is characterized by a wide band gap. The differences in lattice structures of anatase and rutile TiO_2_ cause different densities and electronic band structures; the band gap for rutile is reported to be ~3.00 eV (direct band gap), and for anatase is ~3.20 eV (indirect band gap) [9,16,23,27,28,29]. The anatase crystal structure shows more photocatalytic activity than the rutile structure. It is considered that this is possibly due to anatase’s slightly higher Fermi level, more hydroxyl groups on the surface and lower ability to adsorb oxygen. The amorphous form of TiO_2_ is considered to be practically photocatalytic inactive, because of the facilitated recombination of the photogenerated electron and the hole [10]. However, both our experience and other work with amorphous titania coatings were allowed to state that these systems have potential as photocatalytically active materials [13,29,30]. Keri et al. also observed the clearly detectable photocatalytic effect of amorphous TiO_2_ deposited by the atomic layer deposition method on the SiO_2_ and PMMA [31]. Moreover, Kaura and Singh used the density function theory (DFT) to investigate structural and electronic properties of amorphous TiO_2_ layers, which can be applied in hydrogen production using photocatalytic water splitting [32]. The results of these studies indicate that amorphous systems should be considered as potential photocatalytically active systems. It is important to have in mind that the difference in photocatalytic activity is associated with numerous different coexisting factors, such as specific surface area, pore and crystal size distribution, preparation methods, degree of surface hydroxylation, and the number of defects in the crystal structure [22,30,31,32,33]. 

Our previous works were mainly focused on the estimation of the bioactivity of titania-based materials produced on the surface of titanium or its alloys’ substrates and differing in architecture and structure [34,35,36,37,38]. These materials were synthesized mainly by electrochemical oxidation (titania nanoporous, nanotubular and nanosonge-like coatings (TNT)), and also by direct chemical oxidation (titania nanofibrous coatings (TNF)), without further annealing. This action was deliberate, because we wanted to check the biological activity of systems in which synthesis procedures are not energy-consuming. We thus hoped that their physicochemical properties, bioactivity and cheaper manufacturing methods could be crucial for their potential use in the construction of medical devices.

During investigations of above-mentioned materials, we noticed clear differences in their biological properties, which were related to the differences in their structure and morphology. Taking into account the fact that titania are known photocatalysts, we also set ourselves a goal to check the photocatalytic activity of these materials, wanting to prove whether there is any chance to use this activity for the sterilization process of medical devices made of the abovementioned biomaterials. The possible commercial use of the studied coatings meant that, in the estimation of their photocatalytic activity, the generally recognized ISO standard (ISO:10678:2010) had to be used [39]. This standard assumes the determination of the photocatalytic activity of ceramic surfaces by analysis of the degradation of methylene blue (MB) in aqueous solution when irradiated by ultraviolet (UVA) light [39,40,41,42,43].

## 2. Materials and Methods

### 2.1. Synthesis of TiO_2_ Nanocoatings

The synthesis, and structural and morphological characterization of titania nanotubes (TNT) and nanofiber (TNF) samples, were carried out considering results of our earlier reports [35,36,37].

The method of Ti6Al4V foil surface anodic oxidation was used to produce the first generation TNT coatings (0.3% HF electrolyte solution, *t* = 20 min) using the following potentials: 10 V (TNT10), 20 V (TNT20) and 40 V (TNT40). The produced TNT coatings were stabilized by immersion in acetone for 10 min and drying at 396 K for 1 h [37].

The samples of TNF coatings on the surface of Ti6Al4V substrates were produced using the chemical oxidation method. The surfaces of the substrates were chemically etched in a ca. 5.8 M HCl, then samples were heated in 30% H_2_O_2_ solution at 358 K under a reflux condenser, for different oxidation times; *t* = 4 h (TNF4), 6 h (TNF6) and 10 h (TNF10). 

Different kinds of TNF coatings were also obtained by etching Ti6Al4V substrates’ surfaces in 2 M HF solution for 10 s. In the next step, samples were heated in 30% H_2_O_2_ solution at 358 K for 72 h in an incubator (TNF72).

### 2.2. Characterization of TiO_2_ Nanocoatings

The morphology and cross section of all samples (TNT, TNF) was observed by using a scanning electron microscope with field emission (SEM, Quanta 3D FEG, Huston, TX, USA). The phase composition and crystallinity were determined using X-ray diffraction (PANalytical X’Pert Pro MPD X-ray diffractometer using 370 Cu Kα radiation, PANalytical B.V., Almelo, The Netherlands). The average crystallite size of the samples was estimated using the Scherrer equation of the X’Pert Plus software (Malvern Panalytical Ltd., Malvern, UK). The structure of the produced coatings was also studied using a Raman spectroscopy microscope (RamanMicro 200 PerkinElmer, PerkinElmer Inc., Waltham, MA, USA). Raman scattering was recorded using a laser of wavelength 785 nm, with maximum power 350 mW. Spectra were registered in the 200–3200 cm^−1^ range using a 20 × 0.40/FN22 objective lens and an exposure time of 10 s each time.

### 2.3. Band Gap Characterization

The band gap energy values of the produced samples was determined on the basis of the analysis of diffuse reflectance UV-Vis spectra (UV-VIS-DRS), which were registered between 250 and 700 nm using a Jasco V-750 spectrophotometer (JASCO Deutschland GmbH, Pfungstadt, Germany). The recorded spectra were evaluated in terms of energy band gap values via Spectra ManagerTM CFR software.

### 2.4. Photocatalytic Activity and Kinetic Calculations

The photobleaching properties of TNT and TNF coatings were studied in accordance with ISO 10678:2010 by the degradation of methylene blue (MB, Chempur, Poland) [44]. TNT and TNF samples were preconditioned previously by exposition to UVA light for 28 h in order to decompose any possible remaining organic contaminants. Then, the samples were placed in quartz cuvettes with 3.5 mL of 2 × 10^−5^ M MB ‘conditioning’ solution for 12 h in the dark. This step was required because substrates tend to adsorb dye molecules. After this period, the solution was replaced by the test solution (3.5 mL of 1 × 10^−5^ M MB), and all samples were illuminated with UVA light (315–400 nm, 65 W, 1 mW/cm^2^). The cuvettes were covered with a transparent quartz glass pane. The reaction solution was stirred every 20 min. The changes in MB concentration (absorbance measured at λ = 664 nm) were registered for 3 h spectrophotometrically (Metertech SP-830 PLUS, Metertech, Inc., Taipei, Taiwan), using 10 mm quartz cuvettes. The kinetics of MB degradation, with the use of titania samples photoinduced by UVA light, was evaluated using the Langmuir-Hinshelwood reaction mechanism, as described in [12,13]. The process efficiency was calculated as the percentage of the dye color removal, using the following equation:

(1) $$\%\;\mathrm{MB}\;\mathrm{decolorization}=\frac{C_{0}-C_{t}}{C_{0}}\times 100$$

where C_0_ is an initial concentration of MB and C_t_ is a dye concentration at a given time *t* [12].

## 3. Results

### 3.1. Morphological and Structural Characterization of the Studied Titania Nanocoatings

**Figure 1:**
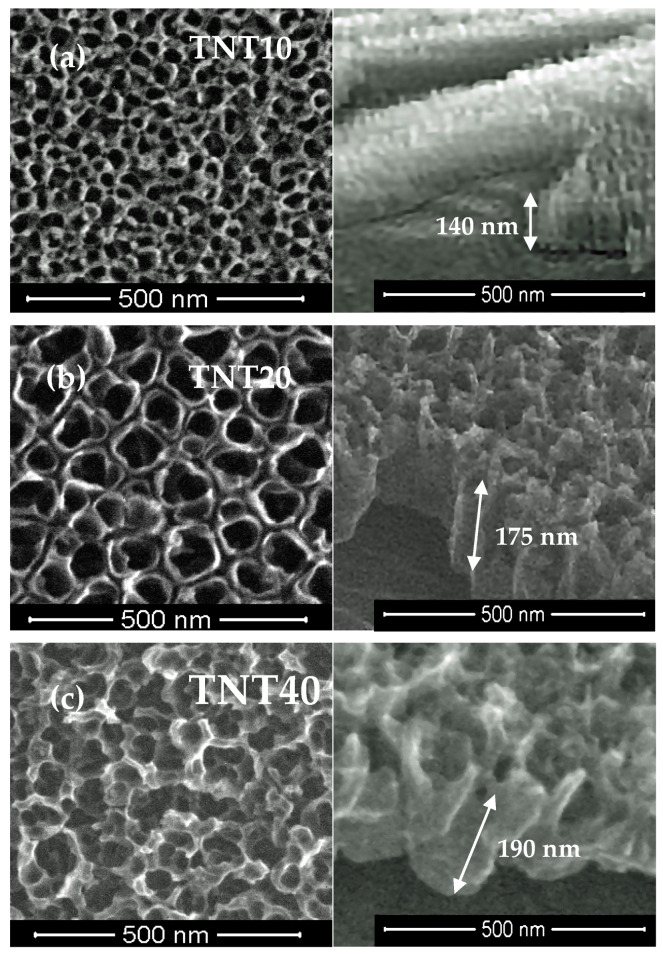
SEM images (top view and cross section) of the nanoporous coating (TNT10 (**a**)), the nanotubular coating (TNT20 (**b**)), and the nanosponge-like system (TNT40 (**c**)) on the surface of the Ti6Al4V alloy samples.

The anodic oxidation method allowed the production of titania nanoporous coatings (TNT10), nanotubular coatings (TNT20) and nanosponge-like coatings (TNT40) dependent on the used potential [35,36]. The morphology and cross-section differences between these three types of TNT systems are presented in Figure 1a–c.

**Figure 2:**
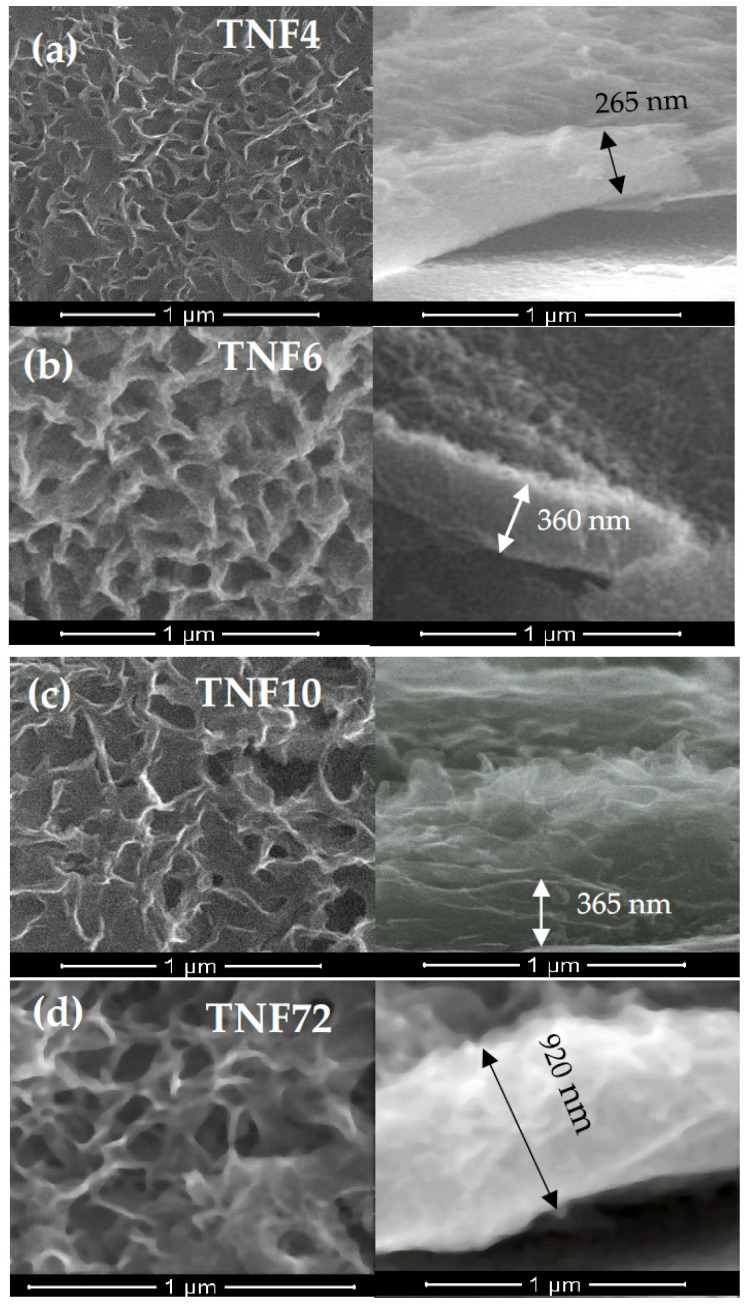
SEM images (top view and cross section) of the surface morphology and cross-sections of titania nanofibers (TNF) produced on the Ti6Al4V substrates: (**a**) TNF4, (**b**) TNF6, (**c**) TNF10, and (**d**) TNF72.

Analysis of the SEM images proved the formation of uniform coatings of the same tube length (approximately 140–190 nm), without cracks or gaps. The XRD and Raman scattering investigations confirmed the amorphousness of all of the produced TNT coatings, which is in agreement with the results of our previous widely run studies of these systems [35,36]. It should be noted that the studied TNT coatings preserved amorphousness up to 553 K, which was confirmed by the results of our earlier investigations [45]. In the synthesis of TNF coatings, 30% H_2_O_2_ solution was used as an oxidation agent, and the oxidation process was carried out at 358 K [37]. We focused on impact studies of the oxidation time (t = 4, 6, 10 h) and a preparation method of the substrate surface (5.8 M HCl and 2 M HF) on the structure and morphology of produced titania layers, and thus on their photocatalytic activity. The direct oxidation of Ti6Al4V samples etched in ca. 5.8 M HCl solution led to the production of fibrous coatings and uniformLy coated substrate surfaces without cracks or gaps (Figure 2a–c).

The use of the other etching method, i.e., immersion of substrate samples in 2 M HF and extending the oxidation time to 72 h, caused the rapid thickness increase of the TNF coatings (ca. 920 nm for TNF72) (Figure 2d).

**Figure 3:**
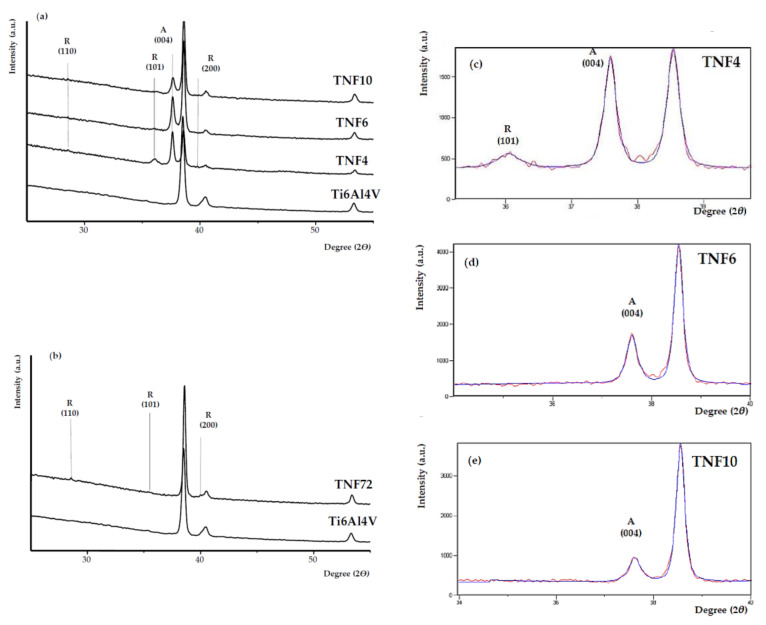
XRD patterns of TNF4–10 (**a**) and TNF72 (**b**) samples, and the Ti6Al4V substrate pattern as a reference sample (TiO_2_ anatase phase (A) and rutile example (R)). Changes of the A(004) and R(101) peaks’ intensities of the TNF4–10 samples, showing the broadening and reduction in intensity at different oxidations time t = 4 h (**c**), 6 h (**d**) and 10 h (**e**).

The X-ray diffraction (XRD) pattern analysis of the TNF4–10 samples (Figure 3a) proved the presence of anatase/rutile phase domains in TNF4 (Figure 3c), and anatase examples in TNF6 and TNF10 (Figure 3d,e) [46].

The average size of the anatase (A) and rutile (R) crystal domains was determined using Scherrer’s equation [47]. Domain sizes, estimated from the (101) R and (004) A peaks, were 27 nm (R), 49 nm (A) for TNF4, 40 nm (A) for TNF6 and 37 nm (A) for TNF10, respectively. Analysis of XRD data revealed the presence of rutile domain traces in the TNF72 sample (Figure 3b). In this case, the domains’ size estimation was not possible due to the weak intensity of the (110)R, (101)R and (200)R XRD peaks.

**Figure 4:**
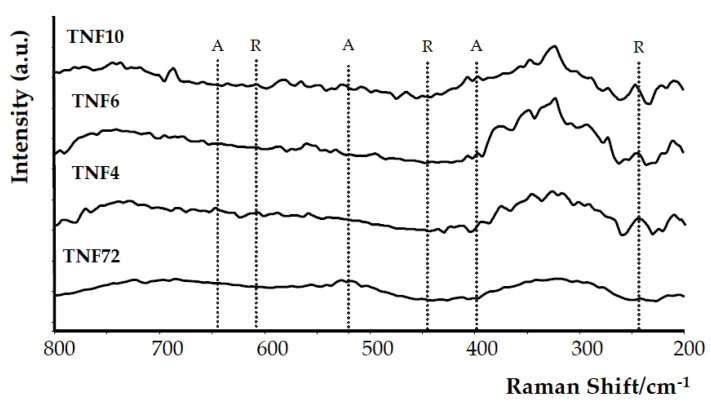
Raman spectra of studied TNF samples (the dashed line indicates the potential positions of the bands derived from the TiO_2_ anatase phase (A) and rutile phase (R)).

Analysis of the Raman spectra registered for TNF4–10 and TNF72 samples revealed that the clear bands which can be assigned to TiO_2_ anatase (398, 516, 640 cm^−1^ [13,48]) or rutile (445, 610 cm^−1^ [13,49]) were not found (Figure 4). Analyzing the XRD patterns and Raman scattering spectra of TNF coatings, one the differences between the received results was observed. Although Raman spectroscopy is considered a more sensitive method for the structural characterization of titanium oxide materials, in this case the more accurate information was provided by the XRD data. This is closely related with a strict dependence between Raman band intensity and the size of crystallites estimated on the basis of the XRD pattern [50]. Ekoi et al. showed that, in the Raman spectra of TiO_2_ layers, which consisted of 76 nm size crystallites, the band intensity was very weak (close to zero) [50]. In the case of the TNF72 system, we were not able to estimate crystallites’ sizes from the XRD spectrum, because it was too low, while for TNF4–10 systems, the estimated crystallite sizes were from 37 to 49 nm. These values were below the limit value given by Ekoi et al., and therefore we approached the XRD spectra with greater confidence. Moreover, previous studies have reported Raman spectra of TiO_2_ samples with very small crystal domains (i.e., around 10 nm) exhibiting quantum confinement effect [51,52]. Therefore, in our case the point to clarify is probably not only the dimension of the nanocrystals themselves, but also their low number compared to the remaining amorphous fraction. Considering the results of the structural analysis (XRD and Raman studies) we assumed that the TNF samples are amorphous and that, depending on the synthesis conditions, they can contain the rutile (TNF72), rutile/anatase (TNF4) and anatase (TNF6, TNF10) phase domains.

### 3.2. Band Gap Characterization

**Figure 5:**
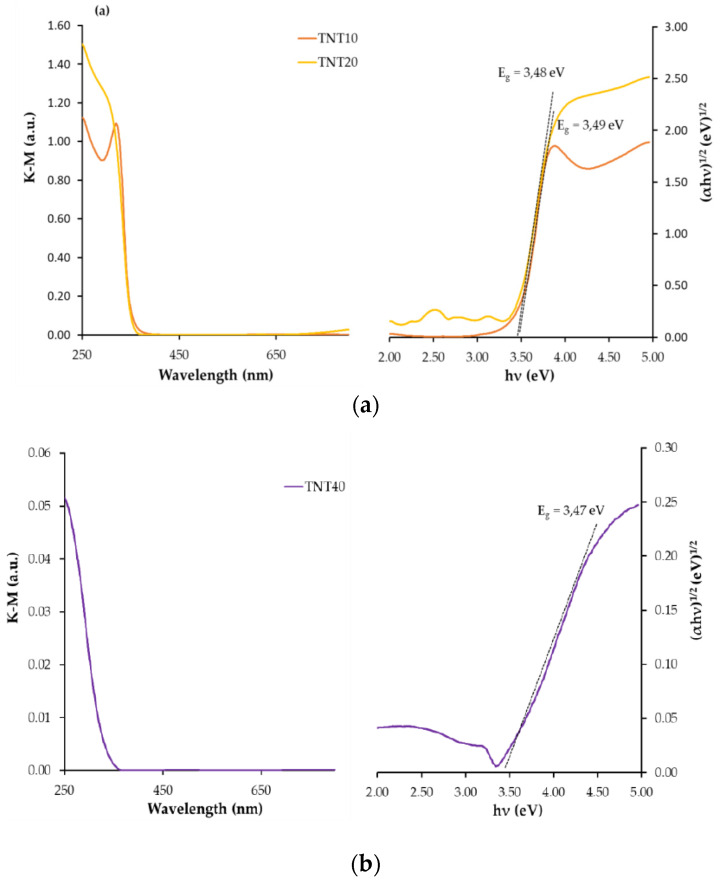
Diffuse reflectance UV-Vis spectra of TNT samples (the left side) and Kubelka–Munk function versus light energy plot for the band gap determination (the right side); (**a**) TNT10 and TNT20 samples and (**b**) TNT40 sample.

**Figure 6:**
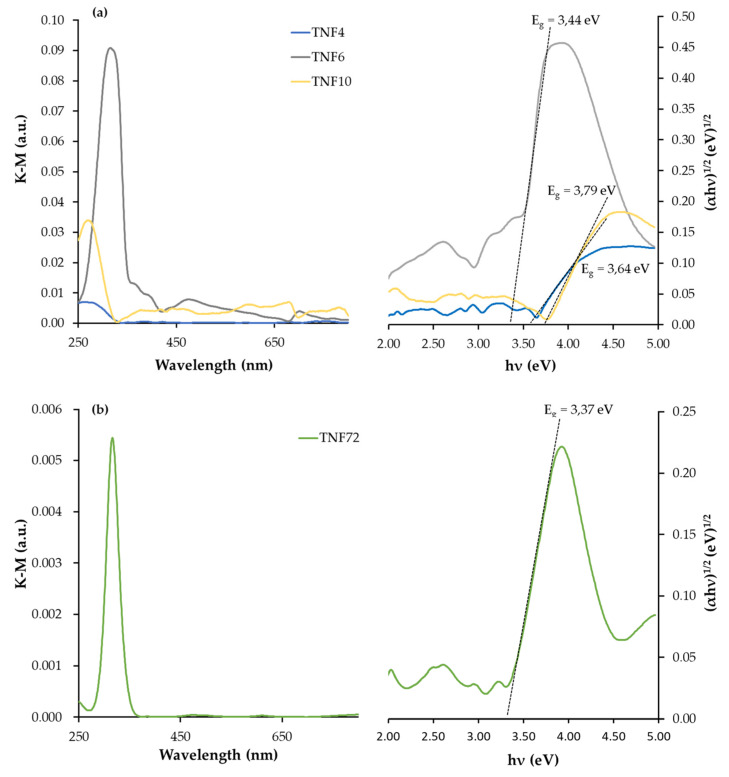
Diffuse reflectance UV-Vis spectra of TNF samples (the left side) and Kubelka–Munk function versus light energy plot for the band gap determination (the right side), (**a**) TNF4–10 and (**b**) TNF72.

The band-gap values (E_BG_) of the studied samples were determined from the UV-VIS-DRS spectrum analysis. The recorded spectra allowed us to make plots of (αhν)^1/2^ vs. hν, with the intercept at (αhν)^1/2^ = 0 for the extrapolated linear part of the plot (Figure 5 and Figure 6). Analysis of data presented in Figure 5 showed that, in the case of amorphous TNT coatings, the E_BG_ values change from 3.47 eV up to 3.49 eV. This points to the lack of significant influence of the TNT coatings architecture, i.e., nanoporous, nanotubular and nanosponge-like systems, on the E_BG_ value (Figure 5).

For TNF specimens with an increase of oxidation time (from 4 h up to 6 h), the E_BG_ values initially decrease from 3.64 eV down to 3.44 eV for TNF4 and TNF6, respectively. Subsequently, the extension of the heating time to 10 h caused the energy values increase up to 3.79 eV (TNF10). By varying the etching conditions of the substrate surface before the oxidation process and extending the time of this process up to 72 h (TNF72), we have obtained a layer characterized by a low E_BG_ value, i.e., 3.37 (Figure 6).

### 3.3. Degradation of MB with the Use of Titania Coatings and Kinetic Calculations

**Figure 7:**
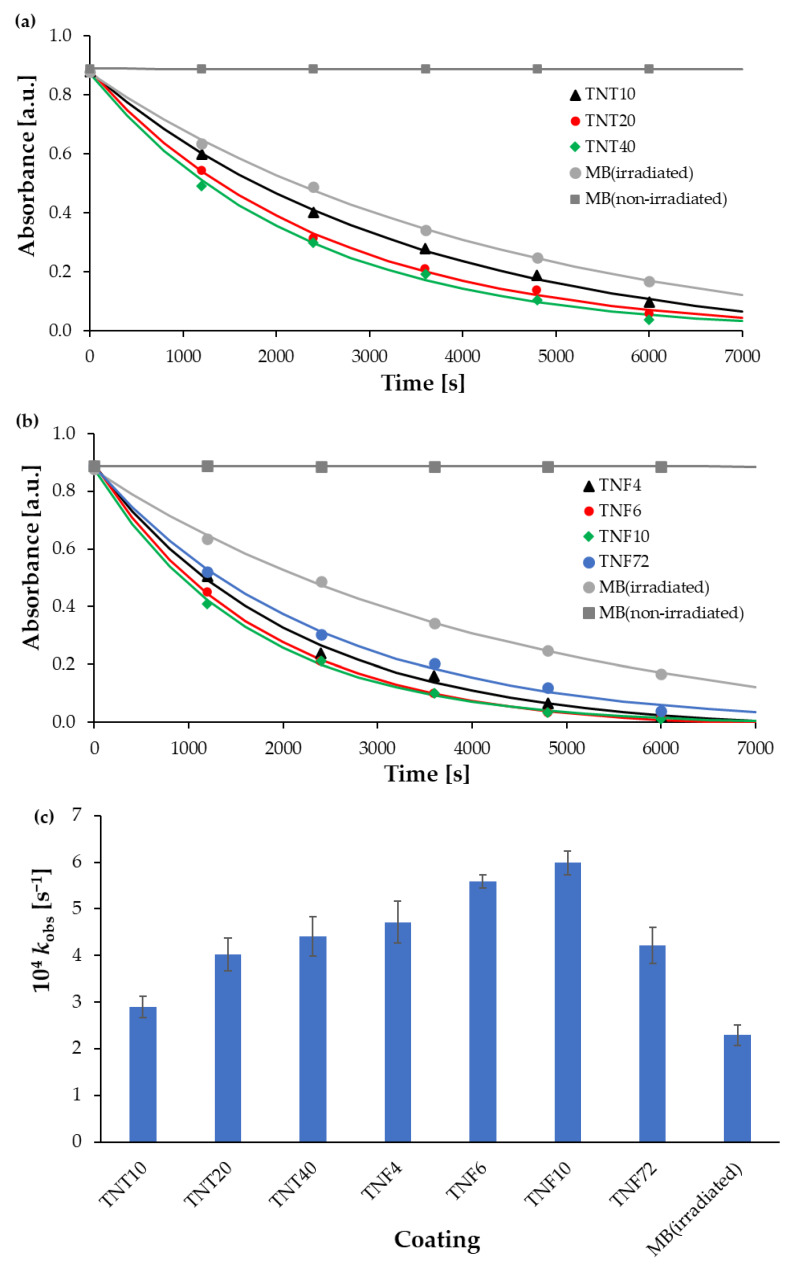
Kinetic runs recorded during MB photocatalytic decolorization with the use of TNT (**a**) and TNF (**b**) samples, and calculated observed rate constants (**c**).

The TiO_2_ coatings on the Ti6Al4V substrate were examined in terms of the decolorization of methylene blue solution. The appropriate observed rate constants (*k*_obs_) for these reactions with TNT and TNF coatings are compared in Table 1 and in Figure 7. According to these data, the photocatalytic decolorization of MB with TNF coatings is a little faster than with TNT coatings. The activity of amorphous TNT coatings and the TNF72 system, which is also amorphous, according to XRD data, but contains the traces of rutile domains, is relatively weak. This can result from the facilitated recombination of the photogenerated electrons and holes in traps on the surface and in the bulk of the particles [26]. In the case of TNF coatings containing polycrystalline domains (TNF4–10) the decolorization percentage and kinetic rate constants increase significantly with the increase of the oxidation time (4–10 h). The application of a UV lamp, ordinarily used to disinfect medical devices, was able to degrade the dye partially, even without titania substrates, as shown in Figure 7. A fast MB degradation process in the presence of photoinduced titania samples allowed us to observe the exponential behavior of the absorbance-time dataset in a time shorter than 100 min.

## 4. Discussion

Due to the fact that, for several years, we have been conducting intensive research on amorphous titania systems with high biocompatibility and application possibilities for the needs of medical devices [34,35,36,37,38], it was important to take a closer look at these systems in terms of checking their photocatalytic activity. Such activity could be used in medical device sterilization processes involving UVA light. The potential commercialization of TNT and TNF coatings imposed a necessity to use the generally recognized ISO standard (ISO: 10678:2010) in the estimation of their photocatalytic activity.

According to literature reports, the structure and the surface morphology are significant factors affecting titania materials’ photocatalytic activity [26,27,28,29,53,54]. Our earlier multiple structural studies of TNT coatings (produced by anodic oxidation) using X-ray diffraction and Raman scattering have proved that, regardless of their surface nanoarchitecture (described as nanoporous (e.g., TNT10), nanotubular (e.g., TNT20) and nanosponge-like (e.g., TNT40)), coatings were amorphous [29]. Analysis of the UV-Vis-DRS spectra of the above mentioned materials showed the lack of significant differences between the energy band gap (E_BG_) values (3.47–3.49 eV) of the studied TNT 10-TNT40 coatings (Figure 5). Studying the photocatalytic activity of the TNT coatings, in accordance with the ISO 10678:2010 standard, we were able to trace the impact of their architecture on their photocatalytic activity more accurately, since the influence of the structure could be excluded (Figure 7 and Table 1). Among the studied TNT samples, TNT40 coatings, i.e., coatings with nanosponge-like architecture, showed the highest photocatalytic activity. Teodorescu-Soare et al. have proved that the photocatalytic activity of nanotube coatings is not only influenced by the materials’ intrinsic characteristics which cause the high charge transfer and low recombination rate of charge carriers, but also depends on morphology and doping [46]. The TiO_2_ nanotube coatings they studied were produced by anodic oxidation using organic electrolytes (glycerol and 0.5 wt.% NH_4_F) and various current densities. The received results proved that the increase in the value of the inner diameter of nanotubes increases the possibility of the penetration of contaminants, and this has a significant impact on the rate of MB decolorization. In our works, in case of amorphous TNT coatings, the fastest decolorization was observed for samples with the biggest tube diameters (TNT40 (120 ± 10 nm)). On the other hand, the results of previous studies exhibited that the values of rate constants depend not only on the surface morphology, but also on the length of the nanotubes [46]. Comparing the results presented for the coatings of tubes with lengths of 0.5 µm, 0.9 µm, 1.4 µm and 2.2 µm, it can be seen that the MB decolorization rate constants increase with the elongation of nanotubes from 1.11 × 10^−5^ s^−1^ through 2.17 × 10^−5^ s^−1^, and 3.92 × 10^−5^ s^−1^ up to 8.08 × 10^−5^ s^−1^, respectively [46]. Amorphous nanoporous, nanotubular and nanosponge-like coatings (TNT10–TNT40), fabricated by us show similar lengths of tubing (140–190 nm) and therefore, in this case, it is rather the surface morphology that plays the most important role in photocatalytic activity. The results of our earlier study of TNT coatings roughness (S_a_) confirm this [34]. From among the studied TNT systems, the largest value of the S_a_ parameter was found for TNT40 (S_a_ = 0.10 µm), while for TNT layers produced at lower potentials the value of this parameter decreased (e.g., for TNT20 S_a_ = 0.58 µm).

Nanofibrous systems (TNF), which were previously checked for biocompatibility [13,36], were subjected to similar tests, aiming at the correlation of structure, morphology and photocatalytic activity. The analysis of their Raman spectra confirmed the amorphous character of the produced TNF coatings. However, the XRD patterns of TNF coatings proved the presence of rutile domains in TNF72, anatase and rutile domains in TNF 4, and anatase domains in TNF6 and TNF10. The mostly amorphous character of a TNF72 coating with a low amount of rutile domains explains its similar photocatalytic activity to a nanosponge-like coating (TNT40, Figure 7c, Table 1). In the case of TNF4–10 samples, analysis of the data presented in Table 1 and Figure 5 showed a clear effect of samples’ surface structures on their photocatalytic activity. The investigated TNF4–10 samples contained the rutile/anatase (TNF4) or mainly anatase phase (TNF6 and TNF10). These coatings have the highest photocatalytic activity among all the studied samples, which can be attributed to the presence of polycrystalline titania forms. We have noticed that systems in which there are mainly anatase domains in the structure (TNF6 and TNF10) were twice as active in the photocatalytic decolorization of MB as amorphous or mainly amorphous ones (TNT, TNF72). Unfortunately, we were unable to find a correlation between the structure of nanofibrous systems and their E_BG_ values, as they changed by leaps and bounds. The obtained results were compared with the observed rate constant (*k*) values of MB decolorization in the presence of TiO_2_ nanoplumes prepared by chemical etching of Ti films in a H_2_O_2_ solution [55]. The reported *k* values changed in the range 8.5 × 10^7^–135 × 10^7^ s^−1^, depending on the oxidation time. According to this report, the use of a longer oxidation time for the titanium substrate in the hydrogen peroxide solution significantly influenced the morphology of the coating and improved its photocatalytic properties. In our study, we observed a similar relation between the time of the oxidation procedure and photocatalytic activity for both of the TNF4–10 samples, but we are more inclined to attribute the increase in photocatalytic activity to the structural changes of the coatings from mixed anatase/rutile systems to systems containing mainly anatase domains.

## 5. Conclusions

The results of the studies carried out allowed us to estimate comparatively, based upon ISO 10678; 2010, the photocatalytic activity of two titania coatings different in morphology and structure which were produced on the surface of Ti6Al4V substrates using the different oxidation methods. The objects of the research were: (a) amorphous TNT coatings of nanoporous, nanotubular and nanosponge-like architectures, produced at room temperature during anodic oxidation; (b) a mostly amorphous TNF72 coating, possessing traces of rutile (R) domains; and (c) TNF samples containing polycrystalline domains (anatase/rutile (A/R), TNF4 and anatase (A) TNF6, TNF10), produced at 358 K by chemical oxidation using 30% H_2_O_2_ solution as an oxidizing agent. 

The photocatalytic activity of the studied titania coatings increases in the order TNT10 (amorphous nanoporous coating) < TNT20 (amorphous nanotubular coating) < TNT40 (amorphous nanosponge-like coating) ≅ TNF72 (mostly amorphous with R domains) < TNF4 (amorphous with A/R domains) < TNF6 (amorphous with A) < TNF10 (amorphous with A). All of the studied coatings, when induced by UVA light in conditions normalized by ISO 10678:2010, degraded MB faster than UVA radiation by itself. Bearing in mind that the tested TNT and TNF coatings showed high biocompatibility in our previous studies, and that they can be used to modify the surfaces of medical devices made of titanium and its alloys, it should be emphasized that, as a result of the tests described in the article, we have additionally proved that the same coatings are able to actively support the sterilization process of medical devices carried out in the presence of UVA radiation, increasing the speed and efficiency of the process of the degradation of organic pollutants.

## Figures and Tables

**Table 1:** Observed rate constants for MB photodegradation (*k*_obs_) on TNT and TNF samples, and MB decolorization percentages.

Sample	10^4^ *k*_obs_ (s^−1^)	MB Decolorization ^a^ (%)
TNT10	2.90 ± 0.23	89
TNT20	4.02 ± 0.35	94
TNT40	4.41 ± 0.42	96
TNF4	4.71 ± 0.45	98
TNF6	5.59 ± 0.14	98
TNF10	5.99 ± 0.26	99
TNF72	4.21 ± 0.38	96
MB (irradiated)	2.29 ± 0.22	81
MB (non-irradiated)	-	0

^a^ MB decolorization at the end of measurements (t = 100 min.)
